# Supplementary material for: P-TRAP: a Panicle Trait Phenotyping tool
Source: BMC Plant Biol. 2013 Aug 29;13:122. doi: 10.1186/1471-2229-13-122 (PMC3848748; doi:10.1186/1471-2229-13-122)
Supplement: Additional file 7 — P-TRAP source code. The Netbeans project that contains the source code of the application. [file 1471-2229-13-122-S7.zip › javadoc.zip!/javadoc/org-bioalg-skeletonprocessor/org/bioalg/skeletonprocessor/GraphProducer.html]

GraphProducer (SkeletonProcessor)


JavaScript is disabled on your browser.


- Package
- Class
- Tree
- Deprecated
- Index
- Help

*org.bioalg.skeletonprocessor 1.0*

- Prev Class
- Next Class

- Frames
- No Frames

- All Classes

- Summary:
- Nested |
- Field |
- Constr |
- Method

- Detail:
- Field |
- Constr |
- Method


org.bioalg.skeletonprocessor

## Class GraphProducer

- java.lang.Object
- - org.bioalg.skeletonprocessor.GraphProducer

- ---

    

  ```
  public abstract class GraphProducer
  extends java.lang.Object
  ```

  Produce a graph from the the image representing it's skeleton

  Author:
  :   faroq

- - ### Constructor Summary

    Constructors

    Constructor and Description

    `GraphProducer()`
  - ### Method Summary

    Methods

    | Modifier and Type | Method and Description |
    | --- | --- |
    | `static org.bioalg.graphprocessor.GraphModel` | `FromImage2GraphModel(java.io.File sourceimage, int smallest_branch, boolean isdarkbackground)` Convert the panicle in the image into a graph. |

    - ### Methods inherited from class java.lang.Object

      `clone, equals, finalize, getClass, hashCode, notify, notifyAll, toString, wait, wait, wait`

- - ### Constructor Detail


    - #### GraphProducer

      ```
      public GraphProducer()
      ```
  - ### Method Detail


    - #### FromImage2GraphModel

      ```
      public static org.bioalg.graphprocessor.GraphModel FromImage2GraphModel(java.io.File sourceimage,
                                                              int smallest_branch,
                                                              boolean isdarkbackground)
      ```

      Convert the panicle in the image into a graph.

      Parameters:
      :   `sourceimage` - source image file path
      :   `smallest_branch` - integer value to be used as a threshold to remove small junk branches

      Returns:
      :   a graph model represent the image `GraphModel`


- Package
- Class
- Tree
- Deprecated
- Index
- Help

*org.bioalg.skeletonprocessor 1.0*

- Prev Class
- Next Class

- Frames
- No Frames

- All Classes

- Summary:
- Nested |
- Field |
- Constr |
- Method

- Detail:
- Field |
- Constr |
- Method
